# Modeling the shape hierarchy for visually guided grasping

**DOI:** 10.3389/fncom.2014.00132

**Published:** 2014-10-27

**Authors:** Omid Rezai, Ashley Kleinhans, Eduardo Matallanas, Ben Selby, Bryan P. Tripp

**Affiliations:** ^1^Department of Systems Design Engineering, Centre for Theoretical Neuroscience, University of WaterlooWaterloo, ON, Canada; ^2^Mobile Intelligent Autonomous Systems, Council for Scientific and Industrial ResearchPretoria, South Africa; ^3^School of Mechanical and Industrial Engineering, University of JohannesburgJohannesburg, South Africa; ^4^ETSI Telecomunicación, Universidad Politécnica de MadridMadrid, Spain

**Keywords:** AIP, CIP, grasping, 3D shape, cosine tuning, superquadrics, Isomap

## Abstract

The monkey anterior intraparietal area (AIP) encodes visual information about three-dimensional object shape that is used to shape the hand for grasping. We modeled shape tuning in visual AIP neurons and its relationship with curvature and gradient information from the caudal intraparietal area (CIP). The main goal was to gain insight into the kinds of shape parameterizations that can account for AIP tuning and that are consistent with both the inputs to AIP and the role of AIP in grasping. We first experimented with superquadric shape parameters. We considered superquadrics because they occupy a role in robotics that is similar to AIP, in that superquadric fits are derived from visual input and used for grasp planning. We also experimented with an alternative shape parameterization that was based on an Isomap dimension reduction of spatial derivatives of depth (i.e., distance from the observer to the object surface). We considered an Isomap-based model because its parameters lacked discontinuities between similar shapes. When we matched the dimension of the Isomap to the number of superquadric parameters, the superquadric model fit the AIP data somewhat more closely. However, higher-dimensional Isomaps provided excellent fits. Also, we found that the Isomap parameters could be approximated much more accurately than superquadric parameters by feedforward neural networks with CIP-like inputs. We conclude that Isomaps, or perhaps alternative dimension reductions of visual inputs to AIP, provide a promising model of AIP electrophysiology data. Further work is needed to test whether such shape parameterizations actually provide an effective basis for grasp control.

## 1. Introduction

The macaque anterior intraparietal area (AIP) receives input from the visual cortex, and is involved in visually guided grasping. A large fraction of neurons in this area encode information about three-dimensional object shapes from visual input (Murata et al., 2000; Sakaguchi et al., 2010). Responses are typically relatively invariant to object position in depth (Srivastava et al., 2009). The responses of some neurons are also invariant to other properties. For example, some are orientation-tuned but not highly sensitive to object shape (Murata et al., 2000). AIP has a strong recurrent connection with premotor area F5, which is involved in hand shaping for grasping (Rizzolatti et al., 1990; Luppino et al., 1999; Borra et al., 2008). Reversible inactivation of AIP leads to grasping impairment, specifically a mismatch between object shape and hand preshape (Gallese et al., 1994; Fogassi et al., 2001). AIP is therefore thought to provide visual information for grasp control (Jeannerod et al., 1995; Fagg and Arbib, 1998).

The focus of this paper is the pathway from V3 and V3A, to the caudal intraparietal area (CIP), to visual-dominant neurons in AIP (Nakamura et al., 2001; Tsutsui et al., 2002). This pathway makes binocular disparity information available for grasp control. Most V3 neurons are selective for binocular disparity (Adams and Zeki, 2001). V3 sends a major projection to V3A (Felleman et al., 1997), which is also strongly activated during binocular disparity processing (Tsao et al., 2003). Both V3 and V3A project to CIP (Katsuyama et al., 2010). CIP neurons are selective for depth gradients (Taira et al., 2000; Tsutsui et al., 2002; Rosenberg et al., 2013) and curvature (Katsuyama et al., 2010). Neurons in AIP receive disynaptic input from V3A via CIP (Nakamura et al., 2001; Borra et al., 2008). Visual-dominant AIP neurons are selective for 3D object shape (Srivastava et al., 2009; Sakaguchi et al., 2010) cued by binocular disparity, consistent with input from this pathway.

AIP also receives many other inputs that we do not model in the present study. The first of these is the premotor area F5, which together with AIP forms a circuit for grasp-related visuomotor transformations. AIP also receives input from the second somatosensory (SII) cortical region (Krubitzer et al., 1995; Fitzgerald et al., 2004; Gregoriou et al., 2006), which may provide tactile feedback and memory-based somatosensory expectations for grasping. Strong connections with other parietal areas are also identified, as well as with prefrontal areas 46 and 12. Area 12 is implicated in high level non-spatial processing including encoding of objects in working memory, suggesting that AIP may be influenced by visual memory of object features (Borra et al., 2008). AIP also contains other neurons that fire in conjunction with motor plans in addition to or instead of visual input (Sakata et al., 1997; Murata et al., 2000; Taira et al., 2000). Interestingly, AIP also receives subcortical input (via the thalamus) from both the cerebellum and basal ganglia (Clower et al., 2005). Finally, AIP receives input from the inferotemporal cortex (IT), which is likely to provide additional visual information about shapes. Our present focus however is the visual input from CIP.

The main goal of this study is to model the neural spike code of object-selective visual-dominant AIP neurons. In particular, we wanted to know whether there are certain sets of shape parameters that are consistent with the responses of visual AIP neurons, and which can furthermore be estimated in a physiologically plausible way from the information available in CIP.

We therefore compared two ways of parameterizing shapes. First we considered the superquadric family of shapes, a continuum that includes cuboids, ellipsoids, spheres, octahedra, and cylinders, and which can also be extended in various ways to model more complex shapes (Solina and Bajcsy, 1990). We considered superquadrics because they play a role in robotic grasp control (Duncan et al., 2013) that seems to be similar to the role of AIP in primate grasp control, i.e., they represent shapes compactly as a basis for grasp planning. We also considered an alternative shape parameterization that is based on non-linear dimension reduction of the depth field. In particular, we used an Isomap (Tenenbaum et al., 2000). We considered Isomap parameters partly because they are continuous, i.e., similar shapes have similar parameters. This is consistent with datasets in which similar 3D stimuli elicit similar spike rate patterns in AIP (Theys et al., 2012, Figure 10; Srivastava et al., 2009, Figure 11C).

This study is one of the first to model the mapping from CIP to AIP. Oztop et al. (2006) modeled AIP as a hidden layer in a multi-layer perceptron network that mapped visual depth onto hand configuration. The output layer of this model (corresponding to F5) was a self-organizing map of subnetworks that corresponded to different hand configurations. Prevete et al. (2011) developed a mixed neural and Gaussian-mixture model in which AIP received monocular infero-temporal input. This model did not include stereoscopic input from CIP. The FARS grasping model (Fagg and Arbib, 1998) did not address in detail how AIP activity arises from visual input. While past AIP models have been relatively abstract, here our goal is to fit published tuning curves from AIP recordings, and furthermore to do so using depth-related input from a model of CIP. As far as we are aware, there have not been previous attempts to model AIP tuning in terms of either superquadric parameters or non-linear dimension reduction of depth features.

## 2. Materials and methods

This study consists of three main parts. The first is a model of tuning for depth features in the caudal intraparietal area (CIP, see Section 2.1.1). The second is a model of tuning for three-dimensional shape features in the anterior intraparietal area (AIP, see Section 2.1.2). Finally, the third is an investigation of physiologically plausible feedforward mappings between CIP and AIP (see Section 2.5).

### 2.1. Cosine-tuning models of neurophysiological data

We tested how well various tuning curves from the CIP and AIP electrophysiology literature could be approximated by cosine-tuned neuron models. In particular, given a vector *x* of stimulus variables, we modeled the net current, *I*, driving spiking activity in each neuron as

(1) $$I={\tilde{\phi }}^{T}x+b,$$

where *b* is a bias term and $\tilde{\phi }$ is parallel to the neuron's preferred direction in the space of stimulus parameters. Longer $\tilde{\phi }$ corresponds to higher sensitivity of the neuron to variations along its preferred direction.

We used a normalized version of the leaky-integrate-and-fire (LIF) spiking model. In this model, the membrane potential *V* has subthreshold dynamics τ_*RC*_ $\dot{V}$ = −*V* + *I*, where τ_*RC*_ is the membrane time constant and *I* is the driving current. The neuron spikes when *V* >= 1, after which *V* is held at 0 for a post-spike refractory time τ_*ref*_ before subthreshold integration begins again. These neurons have spike rate

(2) $$r=\frac{1}{\tau_{ref}-\tau_{RC}\cdot \ln(1-\frac{1}{I})}.$$

Except where noted, τ_*RC*_ was included among the optimization parameters and constrained to the range [0.02*s*, 0.2*s*]. In some cases (where noted), when the basic cosine-LIF model (above) produced poor fits, we also added Gaussian background noise to *I*. Such background noise more realistically reflects the input to neurons *in vivo* (Carandini, 2004) and causes the LIF model to emit more realistic, irregular spike trains. It also has the potential to produce better tuning curve fits. The reason is that depending on the amplitude of the noise, the spike-rate function may be compressive [as in Equation (2)], sigmoidal, or nearly linear. In these cases we fixed τ_*ref*_ = 0.005s and τ_*RC*_ = 0.02*s*, included the noise variance as an optimization parameter, and interpolated the spike rate from a lookup table based on simulations. Given a tuning curve from the electrophysiology literature and a list of hypothesized tuning variables, we found least-squares optimal parameters $\tilde{\phi }$ and *b* mainly, and either τ_*RC*_ or σ_*noise*_ (as noted in the corresponding sections), using Matlab's *lsqcurvefit* function. This function uses Matlab's trust-region-reflective algorithm, which is based partly on Coleman and Li (1994), to solve a non-linear curve-fitting problem in the sense of least-squares. We retried each optimization with at least 1000 random initial points in order to increase the probability of finding a global optimum.

We preferred cosine tuning models over more complex non-linear models for a number of reasons, including that they are simple and that cosine tuning is widespread in the cortex and elsewhere (Zhang and Sejnowski, 1999). (See more detailed rationale in the Discussion).

#### 2.1.1. CIP tuning

We approximated CIP responses in terms of depth and its first and second spatial derivatives. CIP has been proposed to encode these variables (Orban et al., 2006), and they have been the basis for several experimental studies of CIP responses (Sakata et al., 1998; Taira et al., 2000; Tsutsui et al., 2001; Katsuyama et al., 2010; Rosenberg et al., 2013).

We fit cosine-tuned LIF neuron models to tuning curves from Tsutsui et al. (2002) and Rosenberg et al. (2013), and from Katsuyama et al. (2010), in which the stimuli varied in terms of first and second derivatives of depth, respectively. The stimuli in Katsuyama et al. (2010) consisted of curved surfaces with depth

(3) $$z=\frac{1}{2}\left(K_{1}x^{2}+K_{2}y^{2}\right).$$

*K*_1_ and *K*_2_ were varied to produce two levels of “curvedness,”

$$C=\sqrt{\frac{K_{max}^{2}+K_{min}^{2}}{2}}$$

and a range of “shape indices”

$$SI=\frac{2}{\pi }\text{arctan}\frac{K_{max}+K_{min}}{K_{max}-K_{min}},$$

where *K*_*max*_ and *K*_*min*_ are the larger and smaller curvatures along the *x* and *y* axes, respectively.

In terms of the depth *z*, the principal curvature along the *x* axis is

(4) $$K_{x}=\frac{\partial^{2}z/\partial x^{2}}{{\left(1+{\left(\partial z/\partial x\right)}^{2}\right)}^{3/2}}$$

(de Vries et al., 1993). For these stimuli ∂*z*/∂*x* = 0 at the center, and so *K_x_* = ∂^2^*z*/∂*x*^2^.

#### 2.1.2. AIP tuning

**Figure 1:**
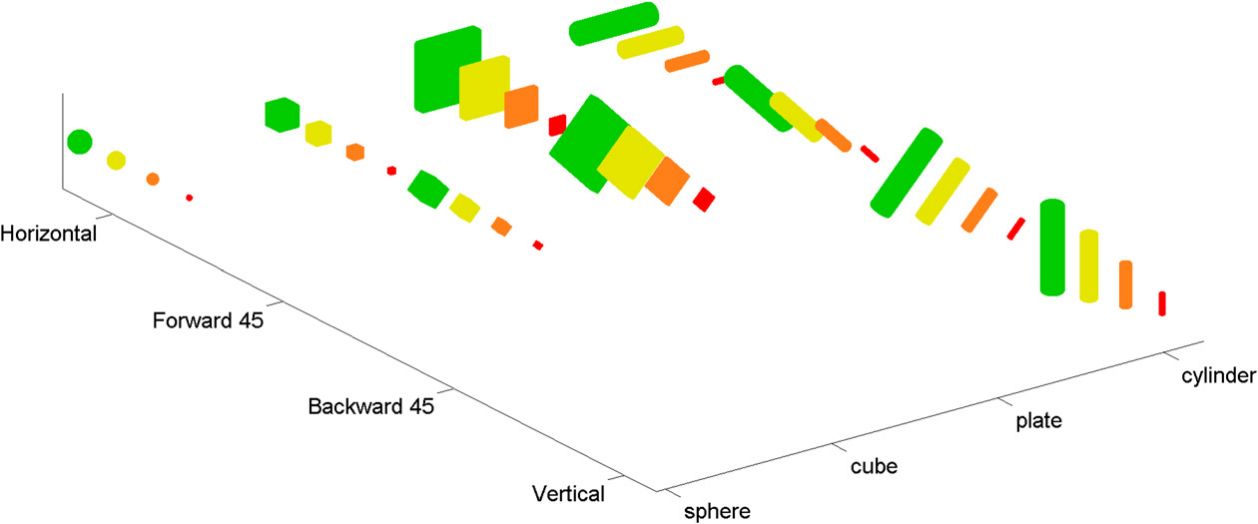
**The complete set of 36 shapes used in the augmented tuning curves**. Four basic shapes (sphere, cube, plate, and cylinder) were adapted from Murata et al. (2000). In order to constrain the models more fully, and in particular to ensure that tuning curves included more points than there were parameters in our models, we augmented these basic shapes by adding copies with different sizes (shown with 4 different colors) and orientations (i.e., horizontal, vertical, tilted forward 45°, tilted backward 45°). Note that due to the symmetry of the basic shapes, some orientations are redundant (e.g., rotating a sphere does not create a distinguishable shape).

**Figure 2:**
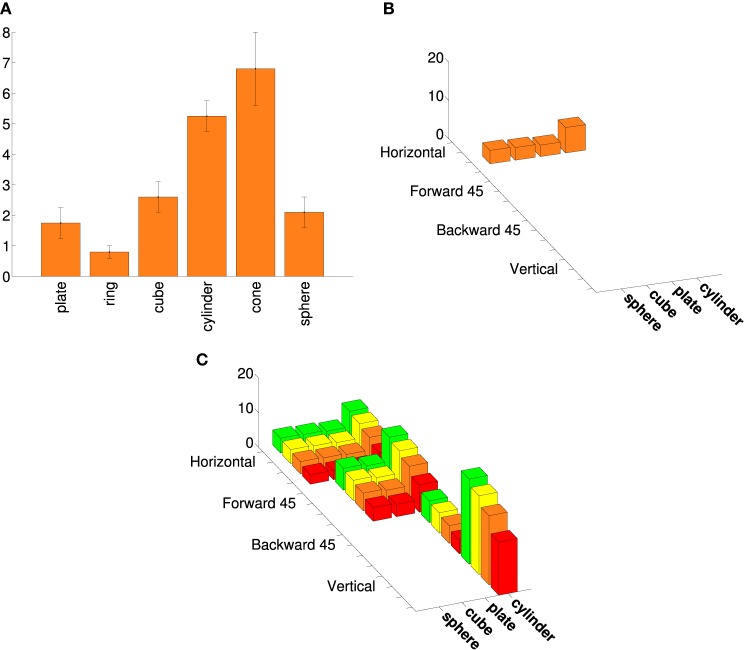
**An example of an augmented AIP tuning curve**. **(A)** Tuning curve adapted from Murata et al. (2000), Selectivity for the shape, size, and orientation of objects for grasping in neurons of monkey parietal area AIP, 2580-2601, with permission. (See their Figure 11.) **(B)** The four points from the same tuning curve that belong to the basic superquadric family (a ring and cone are excluded from the current study). The spike rates are plotted as 3D bars. **(C)** An augmented tuning curve that includes the points in **(B)**, as well as other rotations and scales. This augmented tuning curve is both size-tuned and orientation-tuned, as were the majority of object-type visual neurons in Murata et al. (2000). Another large minority were orientation-tuned but not size-tuned. As in Figure 1, the colors correspond to different sizes.

Following Sakata et al. (1998) and Murata et al. (2000) and consistent with the role of AIP in grasping (Fagg and Arbib, 1998), we took the visual-dominant neurons in AIP to be responsive to three-dimensional shape. Available tuning curves (e.g., Murata et al., 2000) span small numbers of data points relative to the large space of shape variations that are relevant to hand pre-shaping. For this reason we fit models to various “augmented” tuning curves that matched published tuning curves for some shapes, and made assumptions about how these neurons might respond to other shapes (see **Figure 2**). These assumptions were based on additional data for separate AIP neurons (see below). Our augmented tuning curves spanned four of the shapes in Murata et al. (2000), specifically a sphere, cylinder, cube, and plate. Two other shapes (ring and cone) were omitted for simplicity, because they require additional superquadric shape parameters (see Section 2.2). The augmented tuning curves spanned four sizes and four orientations for each of the four shapes. Due to symmetries in the shapes, there were a total of 36 points in these tuning curves (see Figure 1). Four of these points corresponded to AIP data, and the rest (the augmented points) were extrapolated from the data.

We based the augmented points on additional data from other AIP neurons, including aggregate data. Murata et al. (2000) provide shape-tuning curves for six different object-type visual-dominant AIP neurons. We tested different augmented versions of these curves with various combinations of size and orientation tuning (see Figure 2). Murata et al. (2000) reported (without plotting shape tuning for these neurons) that most object-type neurons were orientation selective, and that 16/26 were size-selective. Therefore, we created two augmented tuning curves for each of the six shape-tuning curves. Both were orientation-selective; one was size-selective and the other was size-invariant. For the size-selective tuning curves we assumed that spike rate increased monotonically with size (consistent with Murata et al., 2000, Figure 19; note that preference for intermediate sizes was reported only for motor-dominant neurons). We assumed that orientation tuning was roughly Gaussian and fairly narrow (consistent with Murata et al., 2000, Figure 18). Some AIP neurons are orientation selective with only mild selectivity across various elongated shapes (Sakata et al., 1998). Therefore, we created a final augmented tuning curve that was orientation selective but responded equally to cylinders and plates. Figure 1 shows an example of an augmented tuning curve and its relationship to the data. This procedure made the tuning curve optimization more challenging. This was important because even our simple cosine-tuned neuron models had more parameters than the number of points in the published tuning curves (see Section 3). It also allowed us to make use of additional AIP data.

### 2.2. Superquadrics

We modeled AIP shape tuning both on the parameters of the superquadric family of shapes, and on an Isomap dimension reduction of depth features. The superquadric family is a continuum that includes cuboids, ellipsoids, spheres, octahedra, and cylinders as examples. Superquadrics are often used to approximate observed shapes as an intermediate step in robotic grasp control (Ikeuchi and Hebert, 1996; Biegelbauer and Vincze, 2007; Goldfeder et al., 2007; Huebner et al., 2008; Duncan et al., 2013). In this context, superquadric shape parameters are typically estimated from 3D point-cloud data using iterative non-linear optimization methods (Huebner et al., 2008).

Their role in robotics suggests that superquadrics are a plausible model of AIP shape tuning. Specifically, they can be parameterized from visual information and they contain information about an object that is useful as a basis for grasp planning. One goal of the present study was to examine their physiological plausibility more closely, by fitting superquadric-tuned neuron models to AIP tuning curves. The surface of a superquadric shape is defined in *x*−*y*−*z* space as

$${\left(\frac{x}{A_{1}}\right)}^{1/\epsilon_{1}}+{\left(\frac{y}{A_{2}}\right)}^{1/\epsilon_{2}}+{\left(\frac{z}{A_{3}}\right)}^{1/\epsilon_{3}}=0,$$

where *A* > 0 are scale parameters and ϵ > 0 are curvature parameters. Values of ϵ close to zero correspond to squared corners, while values close to one correspond to rounded corners. For example a sphere has *A*_1_ = *A*_2_ = *A*_3_ and ϵ_1_ = ϵ_2_ = ϵ_3_ = 1. We also used another parameter, θ, that described the orientation of the superquadric. θ was composed of three angles, one per coordinate. The rotation of the superquadric is done applying the rotation matrix described in Equation 5.

(5) $$R(\theta_{1},\theta_{2},\theta_{3})=\left[\begin{array}{ccc} \cos(\theta_{2})\cdot \cos(\theta_{3}) & \cos(\theta_{1})\cdot \sin(\theta_{3})+\sin(\theta_{1})\cdot \sin(\theta_{2})\cdot \cos(\theta_{3}) & \sin(\theta_{1})\cdot \sin(\theta_{3})-\cos(\theta_{1})\cdot \sin(\theta_{2})\cdot \cos(\theta_{3})\\ -\cos(\theta_{2})\cdot \sin(\theta_{3}) & \cos(\theta_{1})\cdot \cos(\theta_{3})-\sin(\theta_{1})\cdot \sin(\theta_{2})\cdot \sin(\theta_{3}) & \sin(\theta_{1})\cdot \cos(\theta_{3})+\cos(\theta_{1})\cdot \sin(\theta_{2})\cdot \sin(\theta_{3})\\ \sin(\theta_{2}) & -\sin(\theta_{1})\cdot \cos(\theta_{2}) & \cos(\theta_{1})\cdot \cos(\theta_{2}) \end{array}\right]$$

We generated a database of 40,000 shapes that included spheres, cylinders, plates, and cubes as well as variations on these shapes with different scales in each dimension, and rotated versions of them. Our database contained roughly equal numbers of box-like, sphere-like, and cylinder-like shapes. For round edges we set ϵ = 1. For squared edges we drew ϵ from an exponential distribution that was shifted slightly away from zero, *p* = 10H(ϵ − η) exp(−(ϵ − η)/0.1) with η = 0.01, where *H* is the Heaviside step function. The shift away from 0 (perfectly sharp corners) helped to avoid numerical problems. The objects had widths between 0.02 m and 0.12 m. We also allowed arbitrary rotations in three dimensions (except where symmetry made rotations redundant), so that each shape had a total of nine parameters.

This study considers only the basic superquadric family, which does not include all the shapes for which AIP responses have been reported. However, the basic family can also be extended in various ways to deal with more complex shapes. For example, hyperquadrics introduce asymmetry (Kumar et al., 1995), and trees of superquadrics can be used to approximate complex shapes with arbitrary precision (Goldfeder et al., 2007).

### 2.3. Creation of depth maps

**Figure 3:**
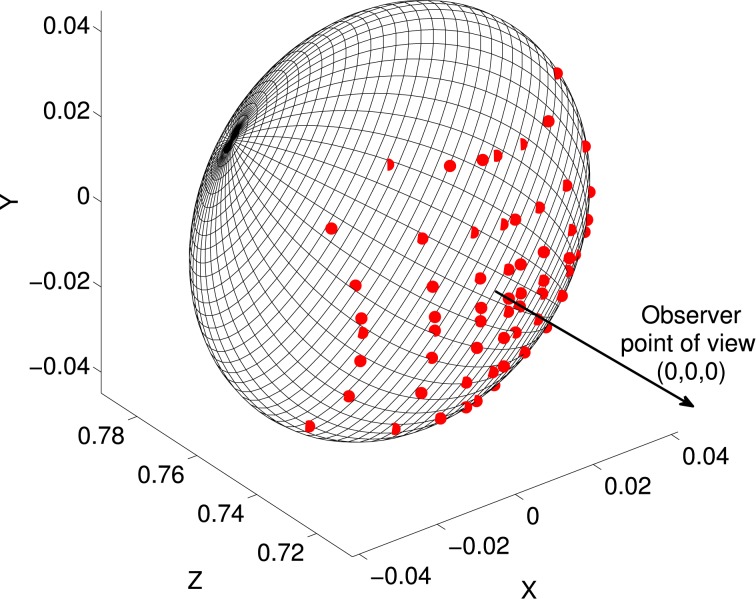
**Illustration of the depth map construction process**. Each superquadric was centered at (0, 0, 0.75) relative to an observer at (0, 0, 0). Rays were traced between the observation point and a grid of points in the frontoparallel plane at *z* = 0.75, and intersections (red dots) were found with the superquadric surface. The depth map consisted of a grid of distances from (0, 0, 0) to these intersections.

CIP receives input from V3 and V3A, which encode binocular disparity information (Anzai et al., 2011). Disparity is monotonically related to visual depth, or distance from observer to surface. As a simplified model of this input we created depth maps, i.e., grids of distances from a viewpoint to object surfaces. We created depth maps from the shapes in our superquadric database by finding intersections of the surfaces with rays at various visual angles from the view point. We used a 16 × 16 grid of visual angles. Grid spacing was closer near the center than in the periphery, in order to reflect higher visual acuity near the fovea and also to ensure that a few rays intersected with the smallest shapes (specifically, distances from the center were *a*^1.5^, where *a* were evenly-spaced points). The grid covered ± 10° of visual angle in each direction. The object centers were at a depth of 0.75 m from the viewpoint. Depth at each grid point was found as the intersection of the superquadric surface with a line from the observation point (Figure 3).

### 2.4. Isomap shape parameters

Within the superquadric family there is typically more than one set of parameters that can describe a given shape. For example, a tall box can either be parameterized as a tall box or a wide box on its end. This is not very problematic in robotics, because an iterative search for matching parameters finds one of these solutions. However, our goal was to model a feedforward mapping from depth (V3A) to shape parameters (AIP). In order to use the superquadric parameters as the basis for an AIP tuning we therefore needed the superquadric-to-depth function to be invertible. We achieved this by restricting the ranges of angles. For example, for box-like shapes we restricted all angles to within ±π/4. This resulted in a unique set of superquadric parameters for each shape. However, large discontinuities remained, in that some very similar shapes sometimes had very different parameters. For example, a tall box at an angle slightly less than π/4 has a depth map that is very similar to a wide box at angle just greater than −π/4 radians. Similar discontinuities seem to exist regardless of the angle convention. We anticipated that these discontinuities would impair feedforward mapping in a neural network, so we also explored an alternative low-dimensional shape parameterization.

In the alternative model, neurons were tuned to an Isomap (Tenenbaum et al., 2000) derived from depth data. Isomap is a non-linear dimension-reduction method in which samples are embedded in a lower-dimensional space in such a way that geodesic distances (i.e., distances along the shortest paths through edges between neighboring points) are maintained as well as possible. This method ensured that similar depth maps would be close together in the shape-parameter space, minimizing parameter discontinuities like those of the superquadric parameters. We constructed an Isomap of the first and second spatial derivatives of the depth maps in the horizontal and vertical directions.

We tested whether our augmented AIP tuning curves (above) were consistent with cosine tuning for these shape parameters. We also tested how well these shape parameters could be approximated by a neural network with CIP parameters as input.

### 2.5. Neural network models of CIP-to-AIP map

In addition to fitting cosine-LIF models to neural tuning curves in CIP and AIP, we also developed feedforward networks to map from CIP variables to AIP variables. Our general approach was to decode shape parameters from the spike rates of CIP models.

We experimented with several different networks including neural engineering framework networks (Eliasmith and Anderson, 2003; Eliasmith et al., 2012), multilayer perceptrons trained with the back-propagation algorithm (Haykin, 1999) and convolutional networks (LeCun et al., 1998).

In each case the output units were linear. Linear decoding of the tuning parameters was of interest because decoding weights can be multiplied with preferred directions to give synaptic weights for any cosine tuning curve over the decoded variables (Eliasmith and Anderson, 2003). Specifically, suppose we have presynaptic rates **r**_*pre*_ and linearly decoded estimates $\hat{p}$ = Φ**r**_*pre*_ of shape parameters **p**, where Φ is a matrix of decoding weights. In this case the family of cosine tuning curves over $\hat{p}$ is

(6) $$r_{post}=G\left({\tilde{\phi }}^{T}\hat{p}+b\right),$$

where ${\tilde{\phi }}^{T}\hat{p}+b$ is the driving current, $\tilde{\phi }$ is the neuron's preferred direction, *G* is a physiological model of the current-spike rate relationship, and *b* is a bias current. Such a tuning curve can then be obtained with synaptic weights (from all *presynaptic* neurons to a single *postsynaptic* neuron)

(7) $$w^{T}={\tilde{\phi }}^{T}\Phi .$$

This allows us to draw general conclusions about how well our various models can account for AIP tuning, and how they would relate to future data.

Equations 6 and 7 are important components of the Neural Engineering Framework (Eliasmith and Anderson, 2003; Eliasmith et al., 2012), a method of developing large-scale neural circuit models.

## 3. Results

### 3.1. CIP tuning

**Figure 4:**
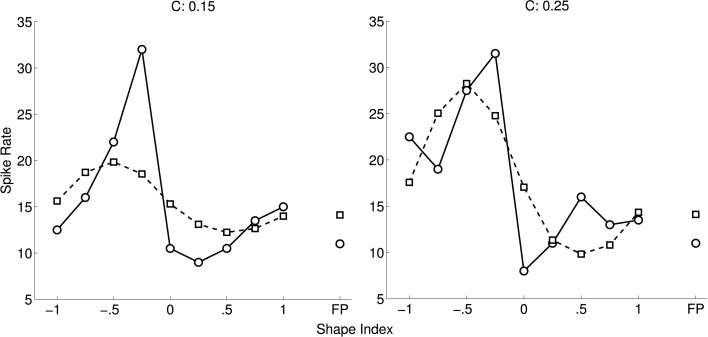
**Fit of CIP model (squares) to tuning curve (circles) of an example neuron (0.04 ± 5.22 spikes/s; mean error ± *SD*)**. The tuning curve is replotted from Katsuyama et al. (2010), with permission from Elsevier. In our model of CIP, neurons are cosine-tuned to five dimensions: depth, horizontal and vertical first spatial derivatives of depth, and horizontal and vertical second spatial derivatives of depth. The stimuli in Katsuyama et al. (2010) varied only in terms of the second derivatives. We also added non-linear tuning functions to improve the fit (see text). The left and right tuning curves are for two different levels of curvedness.

Figure 4 shows an optimal fit of a cosine-tuned LIF model to a tuning curve from Katsuyama et al. (2010). Following their convention the spike rates are shown as a function of shape index, separately for the two curvedness levels. Inspection of the tuning curve revealed that it contained an expansive non-linearity, so we included Gaussian background noise in the model (as described in Section 2). To improve the fit further, in addition to tuning variables *X* = ∂^2^*z*/∂*x*^2^ and *Y* = ∂^2^*z*/∂*y*^2^ we introduced new tuning variables $\frac{1}{2}$(3(*X*)^2^ − 1) and $\frac{1}{2}$(3(*Y*)^2^ − 1). The rationale for their inclusion was that these are the non-linear functions for which linear reconstruction is (with reasonable assumptions) most accurate from populations of LIF neurons tuned to *X* and *Y* (Eliasmith and Anderson, 2003). However, the fit to the Katsuyama et al. (2010) data remained poor despite these measures.

We considered whether a linear-nonlinear receptive field model with depth inputs might produce a better fit. Such models are essentially cosine tuning models with multiple input variables on a grid. However, the depth stimuli in this case (see Equation 3) consisted of linear combinations of *x*^2^ and *y*^2^, so any receptive-field model over the depth field has an equivalent cosine tuning model over *K*_1_ and *K*_2_. Therefore, the neuron is not cosine tuned to either depth or the curvature parameters.

**Figure 5:**
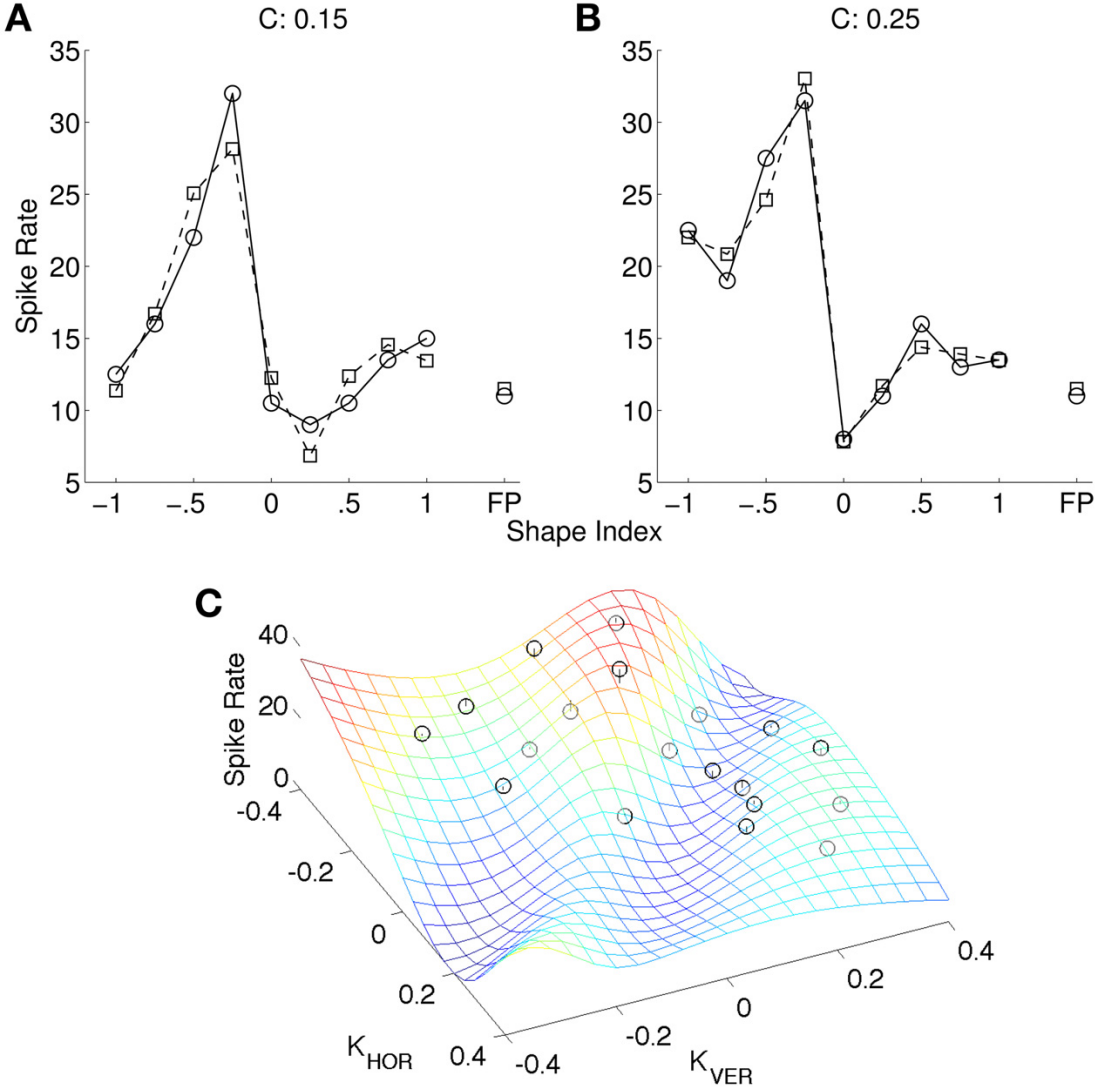
**(A)** Non-linear model (squares) of same neuron as in Figure 4 (circles). **(B)** The same spike rates as **(A)** (black circles), re-plotted as a function of ∂^2^*z*/∂*x*^2^ and ∂^2^*z*/∂*y*^2^, and the best model fit (mesh) (0.00 ± 1.82 spikes/s; mean error ± *SD*). The data plots (black circles) are adapted from Katsuyama et al. (2010), with permission from Elsevier.

Figure 5 shows an example of a more complex non-linear neuron model that fits the data. This model is based on non-linear interactions between nearby inputs on the same dendrite, which suggest that pyramidal cells may function similarly to multilayer perceptrons (Polsky et al., 2004). The input to this model was a 3 × 3 depth grid. The model contained 50 dendritic branches, each of which was cosine tuned to the depths. The linear kernels (analogous to preferred directions) were random. The output of each branch was a sigmoid function of the point-wise product of the depth stimulus and the linear kernel. The spike rate was a least-squares optimal weighted sum of the branch outputs. This was found using a matrix pseudoinverse that used 14 singular values. We also created another version of this model (not shown) in which the tuning curve was augmented with additional stimuli (completing the outer circle of points in Figure 5B) and it was assumed that the neuron would respond to these stimuli at the background spike rate. This version of the model therefore fit 26 points, and we used 20 singular values in the pseudoinverse. The fit was similar in this case.

We also constructed another alternative model of this cell that was based on a more detailed model of V3A activity. Specifically, instead of a 3 × 3 depth grid, this model received input from seven non-linear functions of depth at each point. Five of these were Gaussian functions based on “tuned near,” “tuned zero,” and “tuned far” neurons (Poggio et al., 1988). Two were sigmoidal functions based on “near” and “far” tuning (Poggio et al., 1988). This model (not shown) reproduced the tuning curve somewhat less accurately than the non-linear cell model above. This was the case regardless of minor variations in the set of input tuning functions and their parameters.

**Figure 6:**
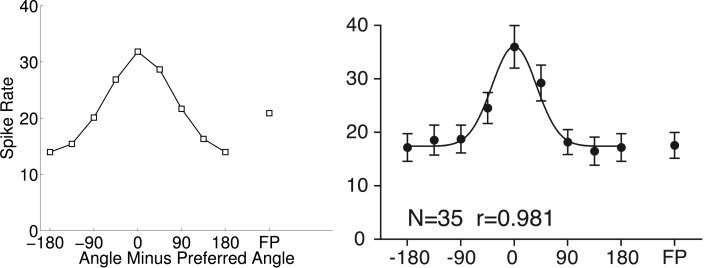
**Cosine tuning model (left) of spike rate data aggregated across neurons (right) (0.03 ± 3.00 spikes/s; mean error ± *SD*)**. The right panel is from Tsutsui et al. (2002). Reprinted with permission from AAAS. In which *N* is the number of neurons and *r* is the regression coefficient.

Figure 6 shows a cosine-tuning fit of data from Tsutsui et al. (2002). This tuning curve is an average over multiple cells that were tuned to depth gradients of visual stimuli. The best fitting cosine-tuning model has a notably different shape than the aggregate data. In particular, the actual spike rates are fairly constant far away from the preferred stimulus, while the model spike rates continue to decrease farther from the preferred stimulus.

Rosenberg et al. (2013) provide several additional CIP tuning curves over 49 different plane stimuli. Some of these tuning curves are clearly not consistent with cosine tuning for first derivatives of depth or disparity, e.g., with multimodal responses to surface tilt. We fit the non-linear model of Figure 5 to seven of these tuning curves (their Figures 4, 5B). Using 20 singular values, the correlations between data and our best model fits were *r* = 0.98 ± 0.01 *SD* for the four tuning curves in their Figure 4, and *r* = 0.78 ± 0.09 *SD* for the three tuning curves in their Figure 5B. (These fits are somewhat closer than fits reported by Rosenberg et al. to Bingham functions, which is unsurprising as our model has more parameters.) Using 40 singular values, our correlations improved to *r* = 0.91 ± 0.02 *SD* for the tuning curves in their Figure 5B.

In summary, the spike rates of these CIP neurons varied with the first and second spatial derivatives of depth, but not in a way that is consistent with cosine tuning to either the depth map, its first and second derivatives, or low-order polynomial functions of these derivatives. Other models, which are physiologically plausible but more complex, fit the data more closely.

### 3.2. AIP tuning

**Figure 7:**
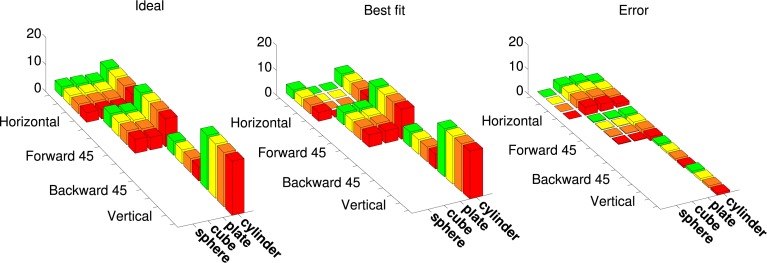
**Best fit of a model neuron that is cosine-tuned over superquadric parameters to an augmented tuning curve**. This augmented tuning curve is size-invariant. Color corresponds to the size of object (see Figure 1) **Left**: Augmented tuning curve. This includes data replotted with permission from Murata et al. (2000). **Center**: Best fit of a cosine-tuned neuron to the augmented tuning curve. **Right**: Error (ideal minus model augmented tuning curve).

Figure 7 shows an example cosine-tuning fit of an augmented tuning curve in superquadric space. This fit is based on a noise-free LIF neuron. For this dataset the shapes were rotated only in one dimension, so we avoided angle discontinuities by using a 2D direction vector in place of the angle. The optimized parameters were the 8-dimensional preferred direction vector $\tilde{\phi }$, the bias *b*, and the membrane time constant τ_*RC*_. Across the 36 points in the augmented tuning curve, the spike rate error (difference between augmented and model spike rates) was 0.70 ± 1.57 (mean ± *SD*).

**Figure 8:**
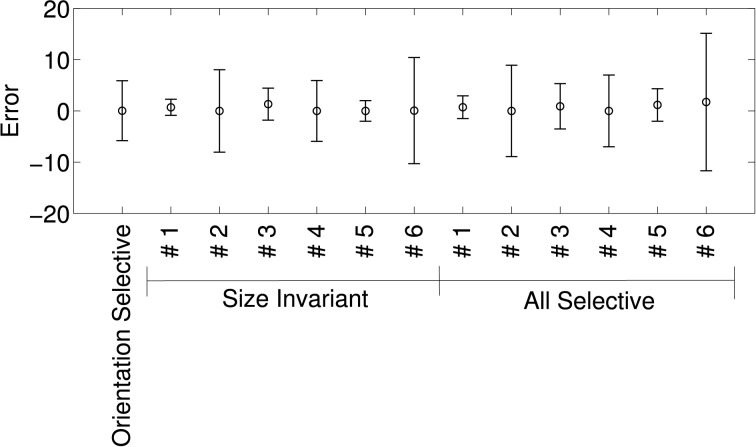
**Quality of fit of cosine tuning model over superquadric parameters with various augmented tuning curves**. The plots shows, for each tuning curve, the mean ± *SD* of the errors over the grid of shapes, sizes, and orientations shown in Figure 7. Note that in this model we can trivially achieve invariance to any superquadric parameter by setting the corresponding component of the preferred direction to zero.

Figure 8 shows the means and standard deviations of spike-rate errors for each of the augmented tuning curves. Good fits were obtained for some of the neurons (#1 and #3 in Murata et al., 2000, Figure 10, and the second in Figure 11, which we label #5). This was true for both size-invariant and size-selective augmented tuning curves. Neuron #1 had low spike rates for the stimuli that we studied. Neurons #3 was highly selective for cylinders, and #5 was more broadly tuned but also preferred cylinders. The worst fits were obtained for neuron #6 which responded strongly to plates and cylinders but not to cubes or spheres.

**Figure 9:**
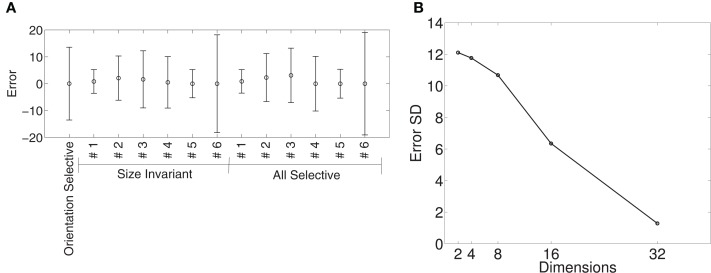
**Quality of fit of cosine tuning model over Isomap parameters with the same augmented tuning curves**. **(A)**, Mean ± *SD* of errors with 8-dimensional Isomap (the same number of parameters as the superquadric family used in Figure 8). Across tuning curves the error is 0.89 ± 10.68. **(B)**, Standard deviation of error over all augmented tuning curves vs. dimension of the Isomap. The error declines sharply with increasing dimension.

Figure 9A shows the means and standard deviations of spike-rate errors for each of the augmented tuning curves in an 8-dimensional Isomap space. We plot the results for the 8-dimensional Isomap in order to match the number of superquadric parameters. The cosine tuning errors (−0.88 ± 10.68 spikes/s; mean ± *SD*) were larger than those in the superquadric space (−0.53 ± 6.75 spikes/s). The difference between these variances was significant according to Levene's test [*W*_(1, 910)_ = 41.3; *p* < 0.001].

Figure 9B shows how the error declined with higher-dimensional Isomaps. Error variance with the 16-dimensional Isomap (−1.77 ± 6.35) was not significantly different from that of the 8-parameter superquadric [Levene's Test; *W*_(1, 910)_ = 1.83; *p* = 0.18]. (Recalculating the variances around 0 instead of −1.77 and −0.89 did not make the difference significant; *p* = 0.058). The cosine-tuning fits were excellent in the 32-dimensional Isomap space, with significantly lower variance [−0.17 ± 1.29 spikes/s; *W*_(1, 910)_ = 316.2; *p* < 0.001]. This higher-dimensional shape representation is therefore consistent with the data and with the augmented tuning curves.

### 3.3. Mapping from CIP to AIP

We trained multi-layer perceptrons in order to understand whether the superquadric or Isomap models of AIP were more consistent with mapping from CIP input. Because CIP neurons are sensitive to depth and to first and second spatial derivatives of depth, we used these as inputs to the networks. Specifically the inputs consisted of 16 × 16 depth maps, their 16 × 16 horizontal and vertical derivatives, and their 16 × 16 horizontal and vertical second derivatives. The derivatives were approximated by convolving with 3 × 3 kernels (e.g., [1 1 1]^*T*^[1 0 −1] and [1 1 1]^*T*^[0.5 − 1 0.5]). The total number of inputs was therefore 16 × 16 × 5 = 1280. The hidden layers had logistic activation functions. The weights and biases were trained with the backpropagation algorithm in Matlab's Neural Network Toolbox. The output layer had a linear activation function in order to model the input to cosine-tuned neurons, as described in the Methods. A dataset of 40000 rotated superquadric objects was generated, from which depth and curvature images were derived. This dataset was divided into 28000 objects for training the network and 12000 objects to validate the results obtained in the training.

**Figure 10:**
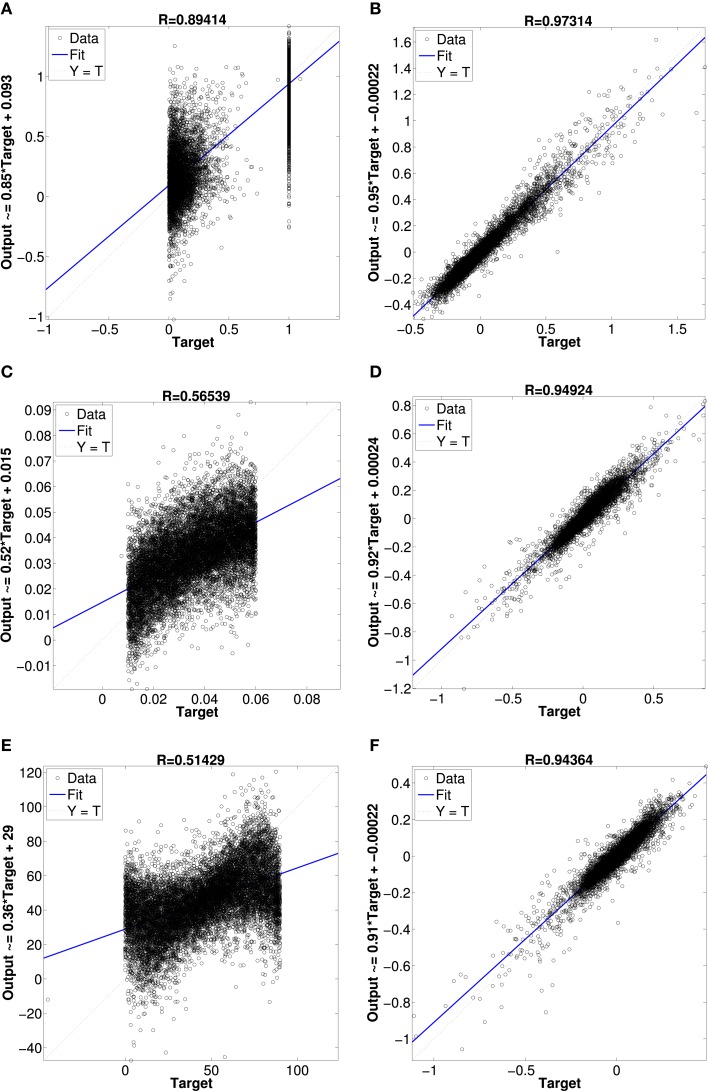
**Regression plot comparison between neural network approximations of superquadrics parameters and Isomap parameters**. **(A)** superquadric epsilon parameter, **(B)** Isomap dimension 1 parameter, **(C)** superquadric scale parameter, **(D)** Isomap dimension 4 parameter, **(E)** superquadric rotation angle parameter and **(F)** Isomap dimension 7 parameter.

Figure 10 shows results from networks with two hidden layers, the first with 600 units and the second with 300 units. The scatter plots show the network's output vs. the actual values of the validation dataset. In Figure 10A is the network's result for the superquadric shape parameter ϵ_1_. The other scatterplots in Figures 10C,E illustrate the network's approximation of the scale and orientation parameters *A*_1_ and θ_1_. Approximation of the other six parameters was similar (e.g., the scatterplots for ϵ_2_ and ϵ_3_ resemble that for ϵ_1_). The scatterplots Figures 10B,D,F illustrate the network's approximation of Isomap parameters. The first, fourth, and seventh dimensions are shown as illustrative examples.

Approximation of the Isomap parameters was much more accurate than approximation of the superquadric parameters. This outcome was very consistent across a variety of networks of different sizes, with one or two hidden layers, with pre-training of hidden layers as autoencoders, etc. We also experimented with networks that contained a hidden layer of LIF neurons with random preferred directions over various local kernels, and optimal linear estimates of the shape parameters from the hidden-layer activity (Eliasmith and Anderson, 2003). The results were also similar in this case, although (as expected) more neurons were required to achieve performance like that of the more fully-optimized multilayer perceptrons.

**Figure 11:**
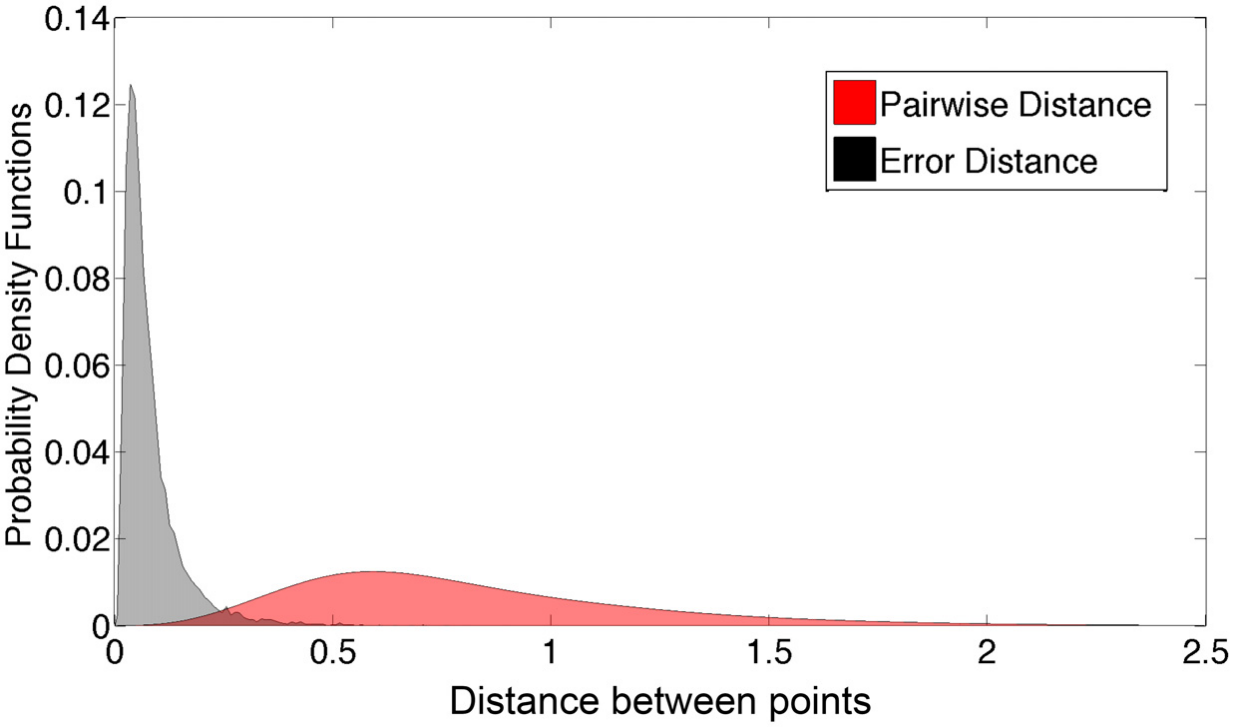
**Histogram of Euclidian distances in Isomap space vs. the root sum square error**.

Figure 11 compares the distribution of the network's Isomap approximation errors with the distribution of pairwise distances between shape examples in our database. The errors were much smaller than typical distances between examples.

We also experimented with a wide variety of larger networks, including convolutional networks, using the cuda-convnet package (Krizhevsky et al., 2012). These networks did not substantially outperform the multilayer perceptron of Figures 10, 11 (lowest mean Euclidean error 0.066 as opposed to 0.081 in Figure 11). We also trained some convolutional networks with only the depth map as input, and with a 3 × 3 kernel in the first convolutional layer. Interestingly, some of the resulting kernels resembled the kernels that we created manually to approximate the first and second derivatives.

## 4. Discussion

This study examined the neural code for three-dimensional shape in visual-dominant AIP neurons. AIP is critical for hand pre-shaping in grasping, and these neurons encode properties that are relevant to grasping including object shape, size, and orientation.

Our motivation for testing superquadric parameters as a model of AIP tuning was that superquadrics have been used in robotics, in a role that we take to be similar to the role of AIP in the primate brain. Specifically, they have been used as compact approximate representations of point clouds on which to base grasp planning. Such a representation is useful because it allows generalization from training examples to unseen examples, e.g., by interpolating between known solutions for known sets of parameters. An alternative approach in robotics is to cluster point clouds into discrete shape categories (Detry et al., 2013). We see the Isomap as an intermediate approach with some of the advantages of both superquadric fitting and clustering. The Isomap is data-driven and adapts to the statistics of the environment (like clustering), but its parameters make up a low-dimensional and continuous space (like those of superquadrics). Furthermore, unlike the superquadric representation, the Isomap representation does not have large discontinuities between very similar shapes.

We found that cosine tuning on a 32-dimensional Isomap accounted well for the tuning curves of object-selective AIP neurons. We also found that, in contrast with superquadric parameters, the Isomap parameters could be approximated fairly well by various neural networks with CIP-like input.

### 4.1. Augmented tuning curves

Available AIP data includes the responses of individual neurons to only a few different shapes, in fact fewer shapes than there are parameters in even the simplest superquadric model. To more vigorously test the different shape parameterizations as a basis for plausible neural tuning, and to incorporate additional aggregate information on shape tuning (e.g., the fact that most visual-dominant AIP neurons are orientation selective), we created “augmented” tuning curves that included both data and extrapolations of the data. It is likely that some of these augmented tuning curves were unrealistic. While the general trends in our AIP fitting results are informative (e.g., that Isomap fits improve and outperform superquadrics as dimensions increase), the details depend on our augmentation assumptions. For example, we found that the Isomap error declined more rapidly when we excluded orientation-selective/shape-invariant tuning curves from the analysis. This limitation does not affect interpretation of our other main result, i.e., that superquadrics were poorly approximated by feedforward neural networks while Isomaps were well approximated.

Future modeling would be facilitated by tuning curves with greater numbers of data points. For example, the dataset in Lehky et al. (2011) includes responses of 674 inferotemporal neurons to a common set of 806 images. A relatively extensive AIP dataset was recently collected (Schaffelhofer and Scherberger, 2014), but no tuning curves from this dataset have yet been published.

### 4.2. Cosine tuning

We were primarily interested in cosine-tuning models for several reasons, not least because cosine tuning is widespread in the brain (see many examples in Zhang and Sejnowski, 1999). Linear-nonlinear receptive field models of the early visual system are another kind of cosine tuning, with multiple tuning variables on a 2D grid. Furthermore, a practical advantage of cosine tuning models is that they require only *n* + 1 tuning parameters for *n* stimulus variables (in contrast a full *n*-dimensional Gaussian tuning curve has *n* + *n*^2^ parameters). This is important because published tuning curves in CIP and AIP consist of relatively few points, so models with large numbers of parameters may be underconstrained. Cosine tuning is also physiologically realistic in that it can arise from linear synaptic integration. For example, if a matrix *W* of synaptic weights has *n* large singular values, then the post-synaptic neurons are tuned to a *n*-dimensional space (if *W* = *U*Σ*V^T^* then the preferred directions are in the first *n* columns of *U*). Cosine tuning curves are also optimal for linear decoding (Salinas and Abbott, 1994). There are also many neurons that do not appear to be cosine tuned, for example speed-tuned neurons in the middle temporal area (Nover et al., 2005). However, where applicable, cosine tuning models provide rich insight into neural activity. We therefore attempted to fit such models to the data where possible. Many AIP tuning curves over similar stimuli with different curvatures vary smoothly and monotonically (Srivastava et al., 2009), consistent with cosine tuning.

Cosine tuning to modest numbers of Isomap parameters (relative to the 256-element depth maps on which they were based) accounted for the AIP data and for our augmented AIP tuning curves.

In contrast, we concluded that the CIP neurons we modeled were not cosine tuned to the stimulus variables with which they have been examined. CIP has been proposed to encode first and second derivatives of depth (Orban et al., 2006). Various neurons in CIP respond to disparity gradient (Shikata et al., 1996; Sakata et al., 1998), texture gradient (Tsutsui et al., 2001), and/or perspective cues for oriented surfaces (Tsutsui et al., 2001). (Accordingly, visual-dominant AIP neurons also respond to monocular visual cues as well as disparity cues, and respond most strongly when disparity and other depth cues are congruent (Romero et al., 2013). Sakata et al. (1998) describe various neurons in CIP as axis-orientation-selective and surface-orientation-selective. The former were sensitive to the orientation of a long cylinder, consistent with two-dimensional tuning for horizontal and vertical curvature. The latter were selective for the orientation of a flat plate, consistent with two-dimensional tuning for depth gradient. Furthermore, Sakata et al. (1998) also recorded a neuron that preferred a cylinder of certain diameter which was tilted back and to the right, but did not respond strongly to a square column of similar dimensions. This suggests selectivity for both first and second derivatives within the same neuron. Katsuyama et al. (2010) recorded CIP responses to curved surfaces that varied in terms of their second derivatives. Tuning to the first and second derivatives of depth is physiologically plausible in that these quantities are linear functions of the depth field, which is available from V3A. We therefore attempted to fit models that were cosine tuned over these variables, but we obtained poor fits.

While CIP neurons are certainly responsive to these variables (and more complex non-linear models of tuning to these variables fit the data closely) it is possible that there are other related variables that provide a more elegant account of these neurons' responses. Notably, some CIP neurons prefer intermediate cylinder diameters (Sakata et al., 1998), whereas cosine tuning for curvature would be constrained to monotonic changes with respect to curvature. Also, some of the neurons in Rosenberg et al. (2013) are clearly non-cosine-tuned for depth slope.

Some CIP tuning curves (see e.g., Figure 6) seem to be fairly similar to rectified cosine functions (Salinas and Abbott, 1994) with a negative offset, except that their baseline rates are not zero. In general, spike sorting limitations, which cannot be completely avoided in extracellular recordings (Harris et al., 2000), are a potential source of uncertainty in tuning curves. However, if misclassification rates had been substantial then multi-peaked tuning curves might have been expected, and none were reported in these studies.

### 4.3. Relationship to shape representation in IT

Area IT has been shown to represent medial axes and surfaces of objects (Yamane et al., 2008; Hung et al., 2012). AIP has significant connections with IT areas including the lower bank of the superior temporal sulcus (STS), specifically areas TEa and TEm (Borra et al., 2008). These areas partially correspond to functional area TEs, which encodes curvature of depth (Janssen et al., 2000) similarly to CIP. However, AIP responds to depth differences much earlier than TEs (Srivastava et al., 2009). It is possible that a shape representation in IT, with some similarities to that in CIP, provides longer latency reinforcement and/or correction of shape representation in AIP.

### 4.4. Future work

A key direction for future work is to test how well the Isomap shape representation works for robotic grasp planning. This would provide important information about the functional plausibility of this representation. For example, if Isomap-based shape parameters cannot be used to shape a hand for effective grasping, this will strongly suggest that there are critical differences between AIP tuning parameters and Isomap parameters. On the other hand, if the Isomap representation performs well, it may suggest a new biologically-inspired approach for robotic grasping.

An apparent advantage of the Isomap approach is that it is data-driven and makes no prior assumptions about shapes. It would be informative to build Isomaps for less idealized shapes that monkeys might grasp in nature.

Other non-linear dimension-reduction methods (e.g., Yan et al., 2007) could also be compared with the Isomap in terms of fitting AIP data and providing an effective basis for grasp planning. We would expect differences relative to Isomap tuning to be subtle relative to available AIP data, but perhaps distinct advantages would appear in a grasp control system. One interesting possibility would be to emphasize features that are related to reward or performance (Bar-Gad et al., 2003).

Another important direction for future work is to extend the model to include motor-dominant AIP neurons and to F5 neurons as in e.g., Theys et al. (2012, 2013) and Raos et al. (2006).

Finally, our models produced constant spike rates in response to static inputs. A more sophisticated future model would account for response timing and dynamics (Sakaguchi et al., 2010). The Neural Engineering Framework (Eliasmith and Anderson, 2003) provides a principled approach to modeling dynamics in systems of spiking neurons.

### Conflict of interest statement

The authors declare that the research was conducted in the absence of any commercial or financial relationships that could be construed as a potential conflict of interest.
